# Enrichment of Aquatic Xylan-Degrading Microbial Communities

**DOI:** 10.3390/microorganisms12081715

**Published:** 2024-08-20

**Authors:** Aline Lucie Odette Gaenssle, Salvador Bertran-Llorens, Peter Joseph Deuss, Edita Jurak

**Affiliations:** 1 Department of Bioproduct Engineering, University of Groningen, Nijenborgh 3, 9747 AG Groningen, The Netherlands; 2 Department of Chemical Engineering, University of Groningen, Nijenborgh 3, 9747 AG Groningen, The Netherlands:

**Keywords:** xylan, environmental sample, microbial enrichment, metagenomic sequencing, brackish water, wheat arabinoxylan (WAX), beechwood glucuronoxylan (BEX)

## Abstract

The transition towards a sustainable society involves the utilization of lignocellulosic biomass as a renewable feedstock for materials, fuel, and base chemicals. Lignocellulose consists of cellulose, hemicellulose, and lignin, forming a complex, recalcitrant matrix where efficient enzymatic saccharification is pivotal for accessing its valuable components. This study investigated microbial communities from brackish Lauwersmeer Lake, in The Netherlands, as a potential source of xylan-degrading enzymes. Environmental sediment samples were enriched with wheat arabinoxylan (WAX) and beechwood glucuronoxylan (BEX), with enrichment on WAX showing higher bacterial growth and complete xylan degradation compared to BEX. Metagenomic sequencing revealed communities consisting almost entirely of bacteria (>99%) and substantial shifts in composition during the enrichment. The first generation of seven-day enrichments on both xylans led to a high accumulation of Gammaproteobacteria (49% WAX, 84% BEX), which were largely replaced by Alphaproteobacteria (42% WAX, 69% BEX) in the fourth generation. Analysis of the protein function within the sequenced genomes showed elevated levels of genes associated with the carbohydrate catabolic process, specifically targeting arabinose, xylose, and xylan, indicating an adaptation to the primary monosaccharides present in the carbon source. The data open up the possibility of discovering novel xylan-degrading proteins from other sources aside from the thoroughly studied Bacteroidota.

## 1. Introduction

To move towards a more sustainable society, it is important to harness the potential of lignocellulosic biomasses as feedstock for materials and fuel production as an alternative to petrol-based sources. Lignocellulosic biomass is generated as a byproduct from agricultural and industrial processes and is the focus of second-generation biorefineries as a more ecological and non-food competitive counterpart to the sugar-rich first-generation biorefineries [1,2]. Its constituents, cellulose, hemicellulose, and lignin, are present in different proportions depending on the type of biomass and species and may even vary throughout the year [3,4].

Cellulose is the most abundant component in the plant cell wall, accounting for 25–45 *w*/*w*%. This polysaccharide is composed of a regular structure made up of (1,4)-β-D-glucosyl units that form microfibrils via hydrogen bonds and van der Waals forces. Additionally, several inter- and intra-molecular forces from the microfibrils generate a crystalline macromolecular structure [5,6], which is more resistant to enzymatic saccharification and protects the plants from bacterial degradation [7].

**Figure 1:**
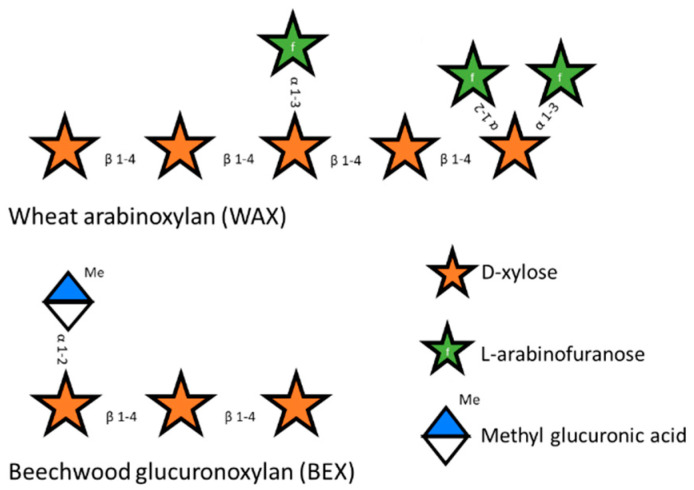
Schematic representation of wheat arabinoxylan (WAX) and beechwood glucuronoxylan (BEX).

Hemicelluloses are more variable than cellulose and, depending on their plant source, can be present as mannan, xyloglucan, xylan, and glucomannan [8]. The most abundant type of hemicellulose is xylan, consisting of xylose residues as a β-1-4 linked backbone that are partly substituted with different groups through various linkages. The main substituent defines the name of the subtype of xylan. Glucoronoxylan is the simplest xylan polysaccharide. It is mostly present in softwoods like birchwood and is characterized by the substitution of the backbone with glucuronic acid and methyl glucuronic acid [9]. Arabinoxylan is mainly substituted by arabinose at the O2 position or at both the O2 and the O3 position and is common in some plants of the *Poaceae* family. Glucuronoarabinoxylan (Figure 1) is a combination of both glucoronoxylan and arabinoxylan and is substituted with glucuronic acid, methyl glucuronic acid, arabinose, and ferulic acid [6,10,11].

The last main polymer in plant cell walls is lignin. Its biosynthesis involves the oxidative polymerization of phenylpropanoid monomers, primarily coniferyl, sinapyl, and *p*-coumaryl alcohols, yielding a heterogeneous and irregular macromolecular network that protects the plant from bacterial attacks by recalcitrance against the enzymatic hydrolysis of plant cell walls [12,13,14]. All three main polymers are also inter- and intra-connected, creating a complex and recalcitrant matrix [9,15,16].

In nature, bacteria have the capacity to hydrolyze xylan, and the different substitutions employ distinct selective enzymes [17]. First, endo-xylanases (EC 3.2.1.8) cleave the main backbone to generate smaller oligosaccharides. The generated fragments are then attacked by accessory xylanolytic enzymes, such as α-l-arabinofuranosidases (EC 3.2.1.55), cleaving the arabinose substitutions, α-glucuronidases (EC 3.2.1.139), removing the linkages between glucuronic acid and xylose, and 1,4-β-xylosidases (EC 3.2.1.37), targeting the bonds between xyloses [17].

Several organisms have been described as capable of degrading plant polysaccharides, including fungi and bacterial taxa such as Actinobacteria, Alphaproteobacteria, Sphingobacteria, and Betaproteobacteria [18]. Aquatic microbial communities may contain such species, as some have been described to degrade grasses [19]. In marine studies, the predominant taxa were typically Pseudomonata [20,21]. In a long-term temperate marine study, Alphaproteobacteria was found to be the most abundant class [21], whereas a study on the communities in an artic fjord identified Gammaproteobacteria to be the predominant class in marine sediment and Flavobacteriales, Verrucomicrobia, and Actinobacteriales in water [20].

The degradation of xylan into monomeric sugars has proved to be a key challenge in using lignocellulosic biomass due to its complex structure [22]. To overcome this limitation of xylan saccharification for a more holistic biorefinery, it is important to find new enzymes capable of xylan degradation. This study aimed to elucidate a possible new source for lignocellulose-degrading bacteria that could contain suitable xylan-degrading enzymes. To date, there have been a large number of studies on microbial communities in the sediment and surface water of both fresh and marine water around the world, but brackish water remains largely understudied. Therefore, the Lauwersmeer in the north of The Netherlands was selected for sampling as it is a lake with a unique environment. Located next to the Wadden Sea and fed by several rivers and canals, it receives both salty and fresh water. The existing gradient of salinity is expected to influence the microbial communities in this environment and may provide a wealthy source for novel lignocellulose-degrading bacteria.

## 2. Material and Methods

### 2.1. Sampling and Site Description

The sampling was conducted in the Lauwersmeer lake in the nature park of Lauwersmeer (Groningen, The Netherlands 53°22′09.2″ N 6°14′17.9″ E), which is in contact with the Wadden Sea in the north (brackish water) and fed with fresh water by canals in the south. Samples were taken in the south from superficial sediment (<10 cm) using two sterile 500 mL bottles and kept at 4 °C during transport and storage.

### 2.2. Substrate Preparation

Beechwood glucuronoxylan (BEX) and wheat arabinoxylan (WAX) were purchased from Megazyme Ltd. (Bray, Ireland). Each substrate was obtained from a single batch. 9.7 g/L substrate solutions of WAX and BEX were prepared by solubilizing the xylan in sterile MQ water with 4.2% ethanol for 20 min at 120 °C. The solution was then added to the medium.

### 2.3. Bacterial Enrichment

The media used for the enrichment was non-reduced Widdel mineral media [23].

The sediment was homogenized and centrifuged at 300 rpm (3 min, 4 °C) to remove soil and vegetation. Then, the bacteria were harvested by centrifugation (10,000 rpm, 15 min, 4 °C) and the cell pellet was washed twice with media before resuspending it in 80 mL media.

For the cultures, 95 mL media (containing 10 g/L xylan) were inoculated with 5 mL cell suspension and incubated in Erlenmeyer flasks for 7 days at 30 °C and shaken at 100 rpm. Three additional generations were grown, which were inoculated with 5 mL of culture from the previous generation. In addition to the closed cultures, open cultures were grown for the first and last generation to conduct daily sampling of 5 mL aliquots.

Both aliquots and cultures were harvested by centrifugation (10,000 rpm, 15 min, 4 °C) and the cell pellet and supernatant were separately flash frozen and stored at −80 °C. For the aliquots, the supernatant was filtered through a 0.22 µm filter before freezing.

During the enrichment, bacterial growth was monitored by measuring the optical density (600 nm) of 100 µL culture in microtiter plates using a spectrophotometer (SpectraMax from Molecular Devices).

All treatments were conducted in duplicates and a negative control (no inoculation) was included in each generation.

### 2.4. Metagenomic Sequencing

Five samples were selected for metagenomic sequencing, being the original sample and after enrichment on either WAX or BEX (1st and 4th generation, closed cultures). The total community DNA was extracted from these samples using the ZymoBIOMICS DNA Miniprep Kit (Zymo Research; Irvine, CA, USA) following the protocol provided by the manufacturer. The purity and quantity of the extracted DNA was verified with a NanoPhotometer (Implen; Munich, Germany; N50) and 1.5% agarose gel.

Metagenomic sequencing on the purified DNA samples was conducted by Eurofins Genomics (Ebersberg, Germany) with the option of bioinformatic analysis “Advance”. The quality of the obtained raw data was first verified by FastQC (bioinformatics.babraham.ac.uk/projects/fastqc/, Version: 0.11.9). The taxonomic distribution was obtained with Kraken 2 [24] (Version 2.1.2) using the standard reference database (github.com/DerrickWood/kraken2, Standard Database, accessed on 23 June 2023) and the samples were combined using KrakenReportAnalyzer (github.com/gaenssle/Kraken_ReportAnalyzer, accessed on 8 August 2023). The functional analysis was conducted with DIAMOND [25] (Version 2.0.13) and all data were visualized using Stata16 (StataCorp; College Station, TX, USA).

### 2.5. Gel Permeation Chromatography

The filtered supernatant from the cultured aliquots (1st and 4th generation) were analyzed with gel permeation chromatography (Agilent Technologies 1200 Series, Santa Clara, CA, USA) with two Suprema PSS columns (100 Å and 1000 Å; 8 × 300 mm 10 µm), tempered at 40 °C and equipped with a refractive index detector. The used eluent was 0.05 M NaNO_3_ and the samples (injection volume 10 µL) were eluted with a flow rate of 1 mg/mL with ethylene glycol as internal standard and a series of 9 pullulan standards (1–708 kDa) as universal standard. Both replicates of the enrichment were analyzed and a representative chromatogram was selected for the graphical representation.

## 3. Results and Discussion

### 3.1. Polysaccharide Degradation and Bacterial Growth

Samples of superficial sediments were collected from a lake with a salinity gradient in The Netherlands and subjected to incubation with two types of xylan: wheat arabinoxylan (WAX) and beechwood glucuronoxylan (BEX). Bacterial growth, estimated through turbidity, polysaccharide degradation, and gel permeation chromatography (GPC), was monitored daily during the first and fourth generations of seven-day enrichment.

**Figure 2:**
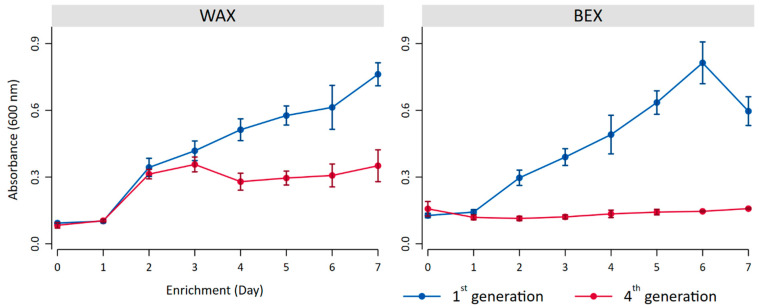
Bacterial growth indicated by the turbidity at 600 nm with BEX and WEX as substrates during the first generation (blue) and the fourth generation of enrichment (red).

During the first generation of enrichment, the bacterial growth exhibited similar patterns for both substrates (Figure 2), demonstrating continuous growth with only a decrease in turbidity for BEX on day 7. It is noteworthy that turbidity served merely as an indicator of growth, as the substrates themselves contribute to turbidity and may fluctuate during degradation processes throughout bacterial growth, potentially explaining the decreased turbidity observed on the final day of BEX incubation.

The fourth generation of enrichment on WAX resulted in initial bacterial growth, followed by stabilization on day 4. Incubation with BEX, on the other hand, showed only low levels of growth, indicating a potential preference of the bacterial community for WAX.

**Figure 3:**
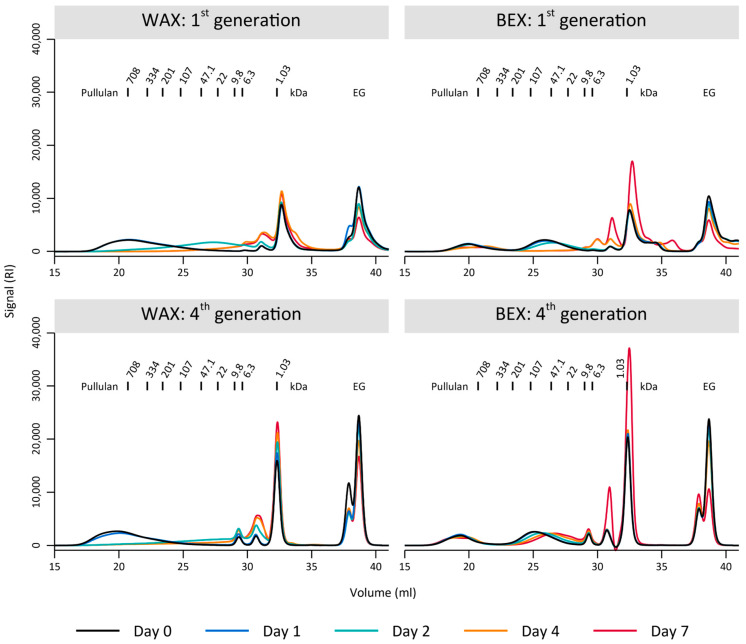
Gel permeation chromatography of the substrates BEX and WAX on several days during the first and fourth generation of 7-day enrichments. EG marks the internal standard ethylene glycol.

The preference of the environmental sample for the WAX substrate was further evident in the polymer degradation observed through GPC (Figure 3). During the initial generation of enrichment, the bacterial culture completely depolymerized the WAX sample, with a population of partly degraded substrate appearing temporarily at day 2 (Figure 3). In contrast, the bacterial culture was unable to fully degrade the BEX, as indicated by the remaining fraction of the initial xylan population between 708–334 kDa, even after 7 days of growth (Figure 3). These differences were even more pronounced after four-week-long generations of enrichment, where WAX was completely degraded after 7 days, with improved depolymerization compared to the first generation (complete degradation at day 2). In contrast, there was only minimal degradation of BEX, notable by the preservation of the largest substrate population between 708–334 kDa and the very limited decrease in the population around 22 kDa.

Therefore, it is plausible that the environmental sample, extracted from an area mostly populated by grass-like plants, was better adapted to degrade WAX, which is more prevalent in such plants compared to glucuronoxylan, which is more commonly found in hardwood biomass [26]. Nevertheless, the loss of the initial BEX degradation and growth is surprising. This could indicate that the bacterial culture was negatively impacted during the different enrichment generations. Some loss of activity could be due to substrate preferences or potential errors during the enrichment period, such as substrate solubilization by adding ethanol, even though this step was conducted for both substrates.

Moreover, the bacterial culture growing with WAX as the only carbon source presented an intriguing culture morphology during the growth. The bacteria in the culture were aggregated in granules (Figure S1), showing the possible production of compounds of interest such as exopolysaccharides [23,27].

### 3.2. Taxonomic Profiling

To study the microbial community responsible for the degradation during the enrichment period, samples were taken after the first generation (WAX1 and BEX1) and fourth generation (WAX4 and BEX4) and subjected to metagenomic sequencing. All four samples resulted in total reads of 14–20 k (Table 1), whereas the original sample (OS) had a lower number of total reads (11 k). Notably, only less than 75% of reads were classified, with under 46% in OS and BEX4. The incomplete classification is most likely due to the reference database used for the identification. The standard database of kraken2 contained about 100 GB of reference genome sequences from Bacteria, Archaea, Viruses, Vectors, and the human genome. As neither the microbial community of the original sample (a brackish lake) nor the enrichment on the substrate (xylan) were studied previously in greater detail, it is plausible that some of the species present in the studied samples were not included in the standard database. Another reason could be the short length of the fragments (150 bp) and the sequencing of the entire genomes. Thus, many fragments could not contain a recognizable marker for the taxonomic analysis.

The determined Shannon Diversity Index ranged from 4.0 to 5.2, and the Equability Index ranged from 0.44 to 0.58. WAX4 had both the highest diversity and evenness, and BEX1 had the lowest values of all samples.

The OS had the highest number of identified species (9.1 k), which reduced to approx. 8.6 k after the first generation of enrichment and to 7.3 k and 6.2 k after the fourth generation for WAX and BEX, respectively. However, only a fraction (<40) were present, with over 20,000 reads (~1% relative abundance).

**Figure 4:**
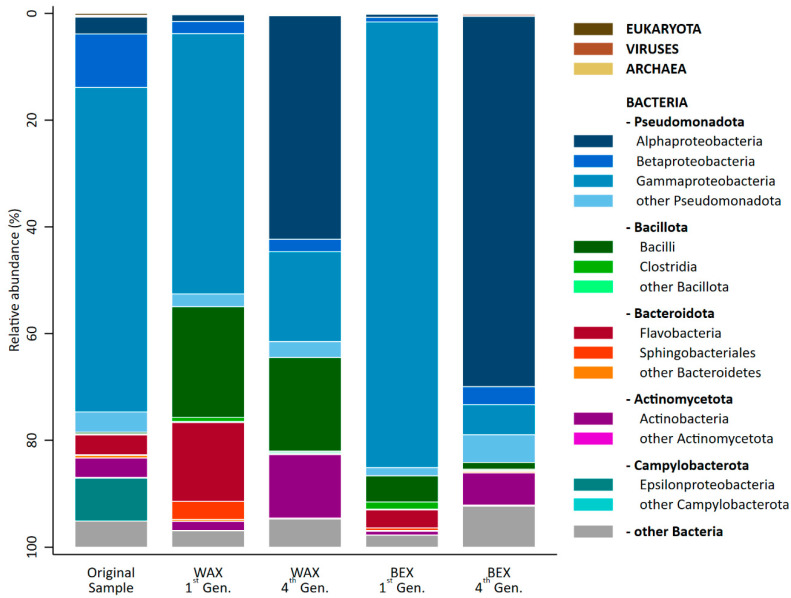
Taxonomic distribution across domains, phyla, and classes of the original sample and the first and fourth generation of enrichment on WAX and BEX. Taxa with fewer than 20,000 reads in all samples were summarized in the categories labelled “other”.

All cultures consisted almost entirely of bacteria (Figure 4), ranging from 99.32% (OS) to 99.86% (BEX1). Additionally, the samples contained traces of Eukaryota (<0.40%), Viruses (<0.30%), and Archaea (<0.20%).

The predominant phylum in all cultures was found to be Pseudomonata, contributing over half of the relative abundance of the microbial community. Notably, samples enriched on BEX contained the highest amount of Pseudomonata (84–87%), followed by the original sample (78%) and the cultures grown in WAX (55–64%). The second-most dominant phylum in the OS was Campylobacteria, which disappeared almost entirely during the xylan enrichment. Enrichment with WAX led to a substantial increase in Bacillota (22% first generation, 18% fourth generation) and a temporary increase in Bacteroidota (19%) in the first generation, which were replaced by Actinomycetota (12%) in the fourth generation. The same phyla and pattern could also be observed for enrichment on BEX, though it was to a lower degree.

All four detected phyla have been described as capable of degrading biomass [18] and are thus in agreement with previous results. Sediment samples from a lake in Groningen (The Netherlands) as well as seawater samples from the southwest coast of India predominantly consisted of Pseudomonata, Bacteroidota, Bacillota, and Actinomycetota [28]. The seawater from the Indian coast further contained Cyanobacteria and Verrucomicrobia [22]. Incubation of the Dutch lake sediment on pre-treated switch grass favored the enrichment of Pseudomonata and Bacillota, with relative abundances changing substantially over time [28]. Furthermore, enrichment studies of the microbial communities on the biomass of various chipped wood piles resulted in an overwhelming majority (>90%) of the species belonging to Actinomycoetota, Pseudomonata, Bacteroidota, and Bacillota [18].

The most apparent change in the microbial community was the dramatic shift (>40%) in both xylan enrichments from Gammaproteobacteria in the original sample and the samples from the first generation to Alphaproteobacteria in the fourth generation. BEX1 consisted almost entirely of Gammaproteobacteria (84%), which were substantially replaced by Alphaproteobacteria (69%).

Gammaproteobacteria usually thrive in anaerobic conditions, such as the sediments of tidal flooded mangrove forests [29], are involved in carbon fixation [22], and occur in response to marine phytoplankton blooms [30]. These blooms lead to the growth of heterotrophic bacteria, classified as Alpha- and Gammaproteobacteria, and Bacteroidota, which have been speculated to perform a division of labor during the degradation of algal-derived organic substrates. While Bacteroidota have been considered to target compounds of high molecular weight, such as proteins and polysaccharides, Gammaproteobacteria were proposed to primarily act as remineralizers of the same polymers, but with less specialization on polysaccharides. Alphaproteobacteria, on the other hand, were generally observed to prefer organic compounds of small molecular weight, such as ethanol, sugar monomers, and amino acids [30]. Thus, even though fresh substrate was supplied at the beginning of each generation, there might have been an accumulation of organic compounds of small molecular weight, favoring an enrichment of Alphaproteobacteria.

Table 2 lists the 20 species with the highest relative abundance. The Actinomycetota *Mycobacterium canetti* was present in all samples. The most abundant species in the original sample were two *Arcobacter* species and *Pseudomona fluorescens*. All other species were only present in traces. Enrichment on WAX led to the accumulation of several Bacillota, as well as two *Cloacibacterium* species and several *Acinetobacter* species. In the fourth generation, almost 8.6% could be attributed to *Niallia circulans*. Incubation with the substrate BEX led to a similar increase in *Acinetobacter* species as well as *Dickeya*. However, in the fourth generation, multiple *Acetobater* and *Komagataeibacter* species were detected.

*Pseudomonas* has been found in multiple environmental sources, associated with lignin degradation, and observed during xylan enrichment [31]. Bacteria belonging to the genus *Bacillus/Niallia* have been described to produce high amounts of xylanases and possess significant xylanase activity [32]. Furthermore, *Enterobacter*, *Acinetobacter*, *Pseudomonas*, *Flavobacterium*, and *Stenotrophonas* are capable of degrading plant lignin, cellulose, and/or hemicellulose [33]. Therefore, the orders found were in good agreement with previous results on microbial biomass degradation. Half of the orders detected in the microbial flora of termite gut were also found in this study, including all three orders of Bacillota (*Bacillales*, *Lactobacillales*, and *Clostridiales*) and three out of four orders of Gammaproteobacteria (*Enterobacterales*, *Pseudomonadales*, and *Xanthomonadales*) [34].

The high amount of *Acetobacter* and other members of the *Acetobacteraceae* family in the fourth generation on BEX was interesting as they are obligate aerobic bacteria adapted to environments rich in sugars and ethanol, such as in the guts of insects with sugar-based diets (e.g., bees and fruitflies) [35]. Analysis of the gut microbiome of the fruitfly *Drosophila melongaster* resulted in a very low diversity, with just five species accounting for 97% of the relative abundance. All five species belonged to just two genera, *Acetobacter* and *Lactobacillus* [36]. Furthermore, the most prominent species (*Acetobacter papayae*, *Acetobacter okinawensis*) have been reported to grow on D-xlyose [37]. Hence, the significant reduction in microbial diversity observed in BEX enrichment, characterized by the selective enrichment of bacteria acclimated to ethanol, notably *Acetobacter*, alongside diminished growth and polymer degradation, suggests a potential loss of enrichment likely attributable to elevated ethanol levels within the BEX enrichment.

### 3.3. Enrichment of Genes Associated with Xylan Degradation and Catabolism

KEGG (kegg.jp) is a database that assigns unique IDs to all of the genomes and genes it includes. Additionally, it assigns IDs to the enzymes encoded in the genes. This enabled an estimation of the occurrence of specific enzymes in specific taxa. Table 3 summarizes the relative occurrence of xylan-specific enzymes present in the genomes of the phyla that were detected in this study (Figure 4).

The three dominant phyla in the original sample were found to only exhibit a low occurrence of the target enzymes. For Campylobacteria, there were no hits for any of the listed enzymes in any genome. This phylum disappeared entirely from the enriched samples. Betaprotebacteria and Gammaproteobacteria, which also only had genomes low on xylan enzymes, decreased in abundance during the enrichment as well. These phyla were replaced by others generally rich in xylan-specific genes. Almost half the genomes of Bacillota were found to contain a xylanase and/or an arabinofurosidase. Genomes classified as Bacteroidota included, on average, one xylosidase, one glycosylceramidase, and several others to a lesser degree. Actinomycetota, found in both enrichments in the fourth generation, had the highest general occurrence of xylanases.

Although the data in Table 3 were not specific for the species found in the samples, the trend towards phyla generally rich in xylan specific genes supported the experimentally obtained results.

A significant portion of carbohydrate-active enzymes (CAZYmes) were identified in bacteria associated with the gut microbiota of various animals, as highlighted by Wardman et al., 2022 [38], with a particular focus on research and identification within the bacterial phylum Bacteroidota [39]. The enrichment of xylan-degrading bacteria from other phyla, such as Pseudomonata, presents a promising opportunity to uncover novel xylan-degrading enzymes.

The hypothesis that the enriched cultures have expressed a sophisticated enzymatic toolkit for the degradation of WAX was supported by the observed increase in genes dedicated to carbohydrate transport and metabolism during the enrichment process (Figure S2). Table 4 highlights the most significant GO (gene orthology) term assignments [40,41], which have been enhanced by over 2000 reads during the enrichment (Table S1). These data demonstrate how the bacterial community, enriched with WAX, has accumulated a higher number of genes associated with the carbohydrate catabolic process (GO:0016052), specifically targeting arabinose (GO:0019568) and xylose/xylan (GO:0045493) catabolic processes. The increased prevalence of these specific catabolic pathways indicates an adaptation to the primary monosaccharides present in the carbon source.

Despite the prominence of genes related to arabinose and xylose catabolism, other carbohydrate catabolic processes have also been enhanced, including those for cellulose (GO:0030245) and arabinan (GO:0031222). Furthermore, the enrichment has led to a significant accumulation of genes associated with specific enzymatic activities, such as xylan 1,4-beta-xylosidase (GO:0009044) and endo-1,4-beta-xylanase (GO:0031176). In addition to hydrolytic activities, there has also been an increase in genes related to carbohydrate binding (GO:0030246).

The analysis of GO terms provides further insights into the predominant degradation and transport mechanisms within the environmental sample. As shown in Table 4, several GO terms are associated with carbohydrate transport (GO:0008643) via ATP-binding cassette (ABC) transporters, including ABC-type D-ribose transporter activity (GO:0015611) and ABC-type L-arabinose transporter activity (GO:0015612). This observation is consistent with previous reports indicating that, in non-Bacteroidota species, ABC transporters are the most common transport mechanism [42,43].

Overall the increased occurrence of xylan-degrading genes associated with the enriched phyla (Table 3), as well as the high enhancement of those in our specific enrichments (Table 4), showcases the adaptation of the enriched culture to use WAX as a carbon source, showing a promising source of non-bacteroidetes carbohydrate-active enzymes.

## 4. Conclusions

In this study, we presented an enrichment of a bacterial community derived from the superficial sediments of a lake characterized by a salinity gradient and an influx of plant material. The environmental bacterial community showed a pronounced and swift adaptation to the substrate, wheat arabinoxylan (WAX), from the onset of the enrichment process. In contrast, beechwood glucuronoxylan (BEX) degradation was nearly lost after four enrichment generations, while WAX degradation was notably enhanced. This trend was further supported by the observed loss of microbial diversity, with the enrichment predominantly favoring Alphaproteobacteria in BEX, while a more diverse and robust culture, including Gammaproteobacteria and Bacillota, was enriched with WAX. Despite the evident bacterial degradation of WAX, supported by optical density (OD), gel permeation chromatography (GPC), and metagenomics analysis, the enriched phyla exhibited only a limited number of known xylan-degrading enzymes, underscoring the potential of the enriched culture as a promising source of novel xylan-degrading enzymes.

## Figures and Tables

**Table 1:** Taxonomic classification of the metagenomic sequencing data.

	OS	WAX1	WAX4	BEX1	BEX4
Total reads	10,924,419	18,135,162	14,350,049	16,357,164	19,858,928
Classified reads (%)	45.32%	61.96%	49.70%	72.67%	27.26%
of which:					
- Classified kingdom (%)	98.33%	99.20%	98.91%	99.17%	95.81%
- Classified species (%)	40.86%	50.56%	50.49%	38.17%	51.38%
Classified species (reads)	2,022,805	5,680,604	3,600,769	4,536,935	2,781,849
Shannon Diversity Index (H)	4.73	4.76	5.23	4.00	4.91
Shannon Equitability Index (E)	0.51	0.52	0.58	0.44	0.56
Species (all)	9095	8592	7258	8557	6241
>100 reads	2175	2565	2883	1259	2057
>5000 reads	51	105	85	66	41
>20,000 reads	12	33	16	27	20

**Table 2:** Overall top 20 species with the highest relative abundance.

Phylum	Class	Species	% OS	% WAX1	% WAX4	% BEX1	% BEX4
Actinomycetota	Actinomycetes	*Mycobacterium canettii*	0.74	0.31	0.41	0.22	2.27
Bacillota	Bacilli	*Exiguobacterium mexicanum*	0.00	1.64	0.00	0.00	0.00
Bacillota	Bacilli	*Leuconostoc mesenteroides*	0.00	1.17	0.00	0.00	0.00
Bacillota	Bacilli	*Niallia circulans*	0.00	4.94	8.55	0.02	0.04
Bacillota	Clostridia	*Clostridium intestinale*	0.00	0.51	0.00	1.14	0.00
Bacteroidota	Flavobacteriia	*Cloacibacterium caeni*	0.00	2.75	0.00	0.32	0.00
Bacteroidota	Flavobacteriia	*Cloacibacterium normanense*	0.00	1.20	0.00	0.15	0.00
Bacteroidota	Sphingobacteriia	*Sphingobacterium hotanense*	0.00	2.10	0.00	0.24	0.00
Campylobacterota	Epsilonproteobacteria	*Arcobacter suis*	1.14	0.00	0.00	0.00	0.00
Campylobacterota	Epsilonproteobacteria	*Arcobacter venerupis*	1.82	0.00	0.00	0.00	0.00
Pseudomonadota	Alphaproteobacteria	*Acetobacter aceti*	0.00	0.00	0.01	0.00	2.42
Pseudomonadota	Alphaproteobacteria	*Acetobacter ghanensis*	0.00	0.00	0.00	0.00	3.91
Pseudomonadota	Alphaproteobacteria	*Acetobacter orientalis*	0.00	0.00	0.00	0.00	1.45
Pseudomonadota	Alphaproteobacteria	*Acetobacter persici*	0.00	0.00	0.00	0.00	2.26
Pseudomonadota	Alphaproteobacteria	*Acetobacter vaccinii*	0.00	0.00	0.00	0.00	5.61
Pseudomonadota	Alphaproteobacteria	*Gluconacetobacter diazotrophicus*	0.00	0.00	0.01	0.00	1.80
Pseudomonadota	Alphaproteobacteria	*Komagataeibacter saccharivorans*	0.00	0.00	0.00	0.00	1.16
Pseudomonadota	Alphaproteobacteria	*Komagataeibacter xylinus*	0.00	0.00	0.00	0.00	1.67
Pseudomonadota	Alphaproteobacteria	*Rhodobacter capsulatus*	0.01	0.01	2.45	0.00	0.03
Pseudomonadota	Alphaproteobacteria	*Rhodobacter sp. LPB0142*	0.00	0.01	3.04	0.00	0.01
Pseudomonadota	Gammaproteobacteria	*Acinetobacter calcoaceticus*	0.01	0.01	1.66	0.03	0.00
Pseudomonadota	Gammaproteobacteria	*Acinetobacter indicus*	0.05	5.26	0.00	2.43	0.01
Pseudomonadota	Gammaproteobacteria	*Acinetobacter radioresistens*	0.02	1.51	0.00	0.08	0.00
Pseudomonadota	Gammaproteobacteria	*Acinetobacter sp. CS-2*	0.20	1.10	0.00	7.19	0.11
Pseudomonadota	Gammaproteobacteria	*Acinetobacter tibetensis*	0.01	0.01	0.00	3.43	0.06
Pseudomonadota	Gammaproteobacteria	*Dickeya oryzae*	0.00	0.00	0.00	1.23	0.00
Pseudomonadota	Gammaproteobacteria	*Dickeya zeae*	0.00	0.00	0.00	2.09	0.00
Pseudomonadota	Gammaproteobacteria	*Pseudomonas fluorescens*	1.78	0.08	0.02	0.04	0.06
Pseudomonadota	Gammaproteobacteria	*Stenotrophomonas maltophilia*	0.02	0.03	2.76	0.31	0.02
Pseudomonadota	Gammaproteobacteria	*Tolumonas auensis*	0.00	0.02	0.00	2.43	0.00

The shown values are colored by their level of relative abundance (white-dark blue).

**Table 3:** Relative occurrence of xylan-specific enzymes in genomes (KEGG database) of relevant phyla.

Enzyme Name	Gene Orthology	KEGG Enzyme	Alpha-Proteob.	Beta-Proteob.	Gamma-Proteob.	Bacillota	Bacter-Oidota	Actino-Mycetota	Campylo-Bacterota
endo-1,4-beta-xylanase	GO:0031176	K01181	24.4%	4.4%	19.5%	35.5%	66.5%	85.0%	0.0%
xylan 1,4-beta-xylosidase	GO:0009044	K01198	35.4%	11.0%	21.6%	48.7%	95.4%	40.4%	0.0%
glucosylceramidase	GO:0004348	K01201	1.5%	2.8%	6.0%	16.0%	91.2%	21.5%	0.0%
alpha-L-arabinofuranosidase	GO:0046556	K01209	25.4%	6.3%	7.4%	45.0%	60.6%	46.0%	0.0%
arabinoxylan arabinofuranohydrolase	GO:0046556	K15921	1.1%	0.0%	0.2%	11.4%	24.3%	6.7%	0.0%
non-reducing end alpha-L-arabinofuranosidase	GO:0046556	K20844	0.4%	1.3%	0.0%	0.0%	0.0%	14.6%	0.0%
arabinan endo-1,5-alpha-L-arabinosidase	GO:0046558	K06113	5.2%	9.4%	7.9%	26.0%	51.1%	26.0%	0.0%
glucuronoarabinoxylan endo-1,4-beta-xylanase	GO:0033940	K15924	0.0%	0.3%	2.3%	8.1%	7.5%	4.5%	0.0%
alpha-glucuronidase	GO:0046559	K01235	5.7%	2.8%	6.6%	11.0%	30.7%	7.9%	0.0%

The data were retrieved on 24 May 2024 using the DomainAnalyzer (github.com/gaenssle/DomainAnalyzer); all shown phyla contained more than 200 genomes and the listed KEGG enzymes had over 200 entries in the shown phyla; The shown values are colored by their level of relative abundance (white-dark blue).

**Table 4:** Most relevant GO terms of which gene occurrence has been increased by at least 2000 reads during the enrichment of the environmental sample with WAX as substrate.

Class	Description	Reads (OS)	Reads (WAX4-OS)	Reads (WAX4-WAX1)
**Carbohydrate metabolism**				
GO:0031222	arabinan catabolic process	259	5779	4595
GO:0046373	L-arabinose metabolic process	266	5181	4231
GO:0045493	xylan catabolic process	953	7667	5584
GO:0019568	arabinose catabolic process	292	2348	2259
GO:0030245	cellulose catabolic process	1163	6683	5314
GO:0042732	D-xylose metabolic process	1112	4424	4040
GO:0016052	carbohydrate catabolic process	1899	4631	1643
GO:0005975	carbohydrate metabolic process	41,423	14,756	9037
**Carbohydrate saccharification**				
GO:0046556	alpha-L-arabinofuranosidase activity	577	8364	6624
GO:0031176	endo-1,4-beta-xylanase activity	291	3241	2487
GO:0004565	beta-galactosidase activity	1074	5758	4123
GO:0009044	xylan 1,4-beta-xylosidase activity	372	2764	1895
GO:0008422	beta-glucosidase activity	1877	5069	941
GO:0030246	carbohydrate binding	7014	12,723	9705
GO:0004553	hydrolase activity, hydrolyzing O-glycosyl compounds	5554	4159	1383
GO:0016787	hydrolase activity	36,954	23,813	15,266
**Carbohydrate transport**				
GO:0015611	ABC-type D-ribose transporter activity	2616	12,337	12,575
GO:0015612	ABC-type L-arabinose transporter activity	928	2416	2652
GO:0042626	ATPase-coupled transmembrane transporter activity	6621	13,762	6725
GO:0055085	transmembrane transport	43,304	80,099	56,900
GO:0140359	ABC-type transporter activity	17,367	23,676	17,570
GO:0055052	ATP-binding cassette (ABC) transporter complex, substrate-binding subunit-containing	43,637	52,747	56,871
GO:0043190	ATP-binding cassette (ABC) transporter complex	30,202	26,827	31,571

The first column shows the reads of the original sample (OS), the second column lists the total difference, and the third column the difference during the first and fourth generation of enrichment on xylan.

## Data Availability

All described data is presented in the manuscript and supplementary materials.

## Supplementary Materials

The following supporting information can be downloaded at: https://www.mdpi.com/article/10.3390/microorganisms12081715/s1, Figure S1: Image of the 7th day of the 4th generation of the culture with WAX as the only carbon source; Figure S2. COG assignment of proteins classified with DIAMOND Table S1: All the GO terms that have been overrepresented more than 2000 reads during the 4 weeks of enrichment with WAX.
