# Supplementary material for: Enrichment of Aquatic Xylan-Degrading Microbial Communities
Source: Microorganisms. 2024 Aug 20;12(8):1715. doi: 10.3390/microorganisms12081715 (PMC11356981; doi:10.3390/microorganisms12081715)
Supplement: Supplementary file 1 [file microorganisms-12-01715-s001.zip › microorganisms-3120570-supplementary.pdf]

Enrichment of aquatic xylan degrading microbial communities

A. L. O. Gaenssle*, S. Bertran-Llorens*, P. J. Deuss, E. Jurak

* These authors contributed equally to this work

Supplementary Information

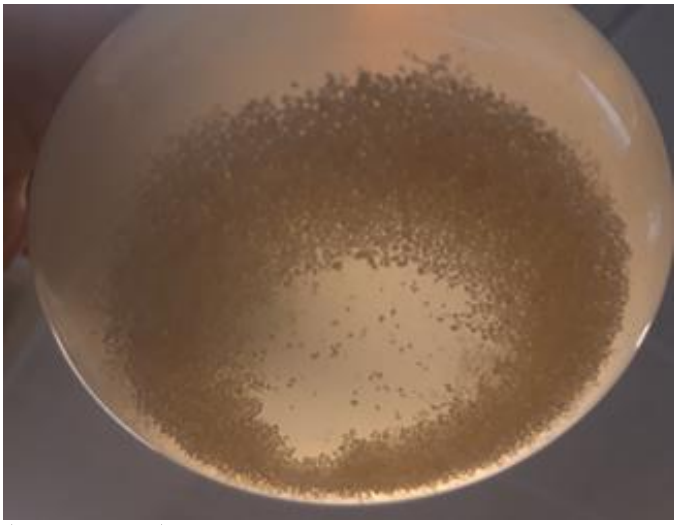

Figure S1. Image of the 7th day of the 4th generation of the culture with WAX as the only carbon source.

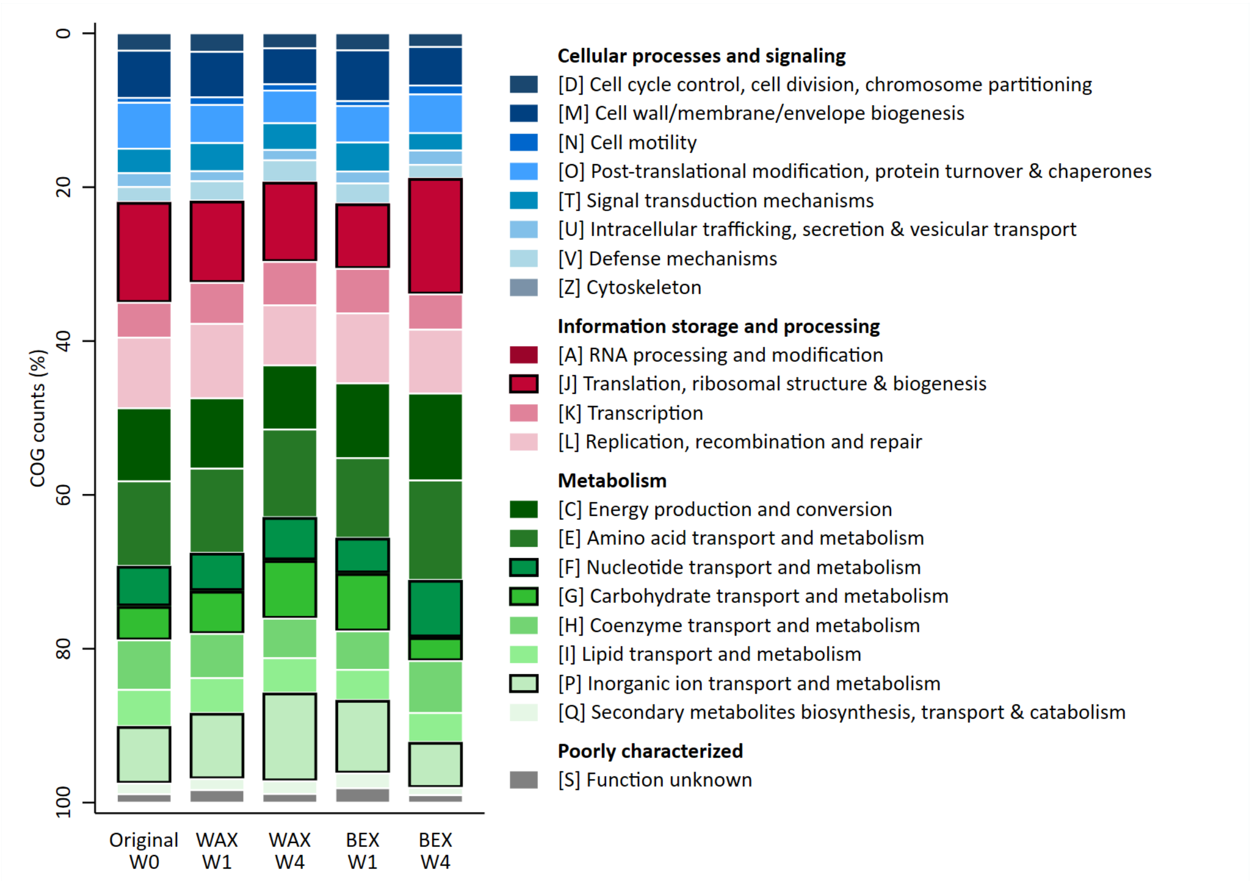

Figure S2. COG assignment of proteins classified with DIAMOND.

Table S1. All the GO terms that have been overrepresented more than 2000 reads during the 4 weeks of enrichment with WAX. The third column represents the reads in the original sample, the 4th row represent the comparison between the original sample and the 4th generation, while the 5th column represents the difference between the 1st and the 4th generation of the enrichment

Class	Description	Original Sample	Difference WAX4-OS	Difference WAX4-WAX1
GO:0042601	endospore-forming forespore	11	3004	648
GO:0018710	acetone carboxylase activity	62	6461	6461
GO:0043443	acetone metabolic process	62	6461	6461
GO:0008706	6-phospho-beta-glucosidase activity	64	3872	705
GO:0103047	methyl beta-D-glucoside 6-phosphate glucohydrolase activity	64	3872	705
GO:0009847	spore germination	355	8903	2541
GO:0031222	arabinan catabolic process	259	5779	4595
GO:0046373	L-arabinose metabolic process	266	5181	4231
GO:0042717	plasma membrane-derived chromatophore membrane	488	8920	9304
GO:0046556	alpha-L-arabinofuranosidase activity	577	8364	6624
GO:0043094	cellular metabolic compound salvage	197	2238	1357
GO:0031176	endo-1,4-beta-xylanase activity	291	3241	2487
GO:0009319	cytochrome o ubiquinol oxidase complex	272	2435	1653
GO:0030698	5,10-methylenetetrahydrofolate-dependent tRNA (m5U54) methyltransferase activity	272	2325	1658
GO:0047151	methylenetetrahydrofolate-tRNA-(uracil-5-)-methyltransferase (FADH2-oxidizing) activity	272	2325	1658

GO:0045493	xylan catabolic process	953	7667	5584
GO:0019568	arabinose catabolic process	292	2348	2259
GO:0052692	raffinose alpha-galactosidase activity	411	3255	2647
GO:0043571	maintenance of CRISPR repeat elements	293	2206	2098
GO:0009044	xylan 1,4-beta-xylosidase activity	372	2764	1895
GO:0035351	heme transmembrane transport	694	4795	4744
GO:0102481	3D-(3,5/4)-trihydroxycyclohexane-1,2-dione hydrolase activity	430	2842	3068
GO:0015453	oxidoreduction-driven active transmembrane transporter activity	370	2432	1691
GO:0009341	beta-galactosidase complex	796	5183	3561
GO:0008982	protein-N(PI)-phosphohistidine-sugar phosphotransferase activity	1,085	6896	-242
GO:0009166	nucleotide catabolic process	376	2308	266
GO:0008880	glucuronate isomerase activity	470	2863	2442
GO:0045733	acetate catabolic process	412	2486	2558
GO:0008663	2',3'-cyclic-nucleotide 2'-phosphodiesterase activity	364	2139	469
GO:0008973	phosphopentomutase activity	493	2877	985
GO:0030245	cellulose catabolic process	1,163	6683	5314
GO:0004565	beta-galactosidase activity	1,074	5758	4123
GO:0047536	2-aminoadipate transaminase activity	394	2096	2225
GO:0043177	organic acid binding	446	2338	1968
GO:0015849	organic acid transport	446	2337	1968
GO:0030435	sporulation resulting in formation of a cellular spore	8,511	44236	-1081
GO:0050538	N-carbamoyl-L-amino-acid hydrolase activity	445	2287	2154
GO:0016813	hydrolase activity, acting on carbon-nitrogen (but not peptide) bonds, in linear amidines	536	2706	2604
GO:0019504	L-proline betaine catabolic process	443	2151	2435
GO:0008810	cellulase activity	435	2075	1636
GO:0090563	protein-phosphocysteine-sugar phosphotransferase activity	969	4607	1166
GO:0015611	ABC-type D-ribose transporter activity	2,616	12337	12575
GO:0016725	oxidoreductase activity, acting on CH or CH2 groups	600	2817	3138
GO:0045121	membrane raft	2,679	12437	-2120
GO:0009264	deoxyribonucleotide catabolic process	612	2819	1301
GO:0050112	inositol 2-dehydrogenase activity	480	2155	2472
GO:0015904	tetracycline transmembrane transport	753	3352	3471
GO:0090452	lithium ion transmembrane transport	753	3352	3471
GO:0033748	hydrogenase (acceptor) activity	1,179	5107	5193
GO:0019310	inositol catabolic process	2,039	8787	9049
GO:0071916	dipeptide transmembrane transporter activity	1,518	6041	6328
GO:1901575	organic substance catabolic process	669	2618	2529
GO:0035725	sodium ion transmembrane transport	957	3714	3423
GO:0042938	dipeptide transport	1,809	6702	7312
GO:0042732	D-xylose metabolic process	1,112	4424	4040
GO:0008643	carbohydrate transport	9,003	31088	27126
GO:0005351	carbohydrate:proton symporter activity	715	2459	1353
GO:0000272	polysaccharide catabolic process	1,149	3850	1042
GO:0009486	cytochrome bo3 ubiquinol oxidase activity	1,762	5087	3882
GO:0006116	NADH oxidation	933	2545	1079
GO:0008422	beta-glucosidase activity	1,877	5069	941
GO:0016682	oxidoreductase activity, acting on diphenols and related substances as donors, oxygen as acceptor	3,228	8631	5808
GO:0033214	siderophore-dependent iron import into cell	1,791	4769	2675
GO:0004368	glycerol-3-phosphate dehydrogenase (quinone) activity	850	2255	1319

GO:0009401	phosphoenolpyruvate-dependent sugar phosphotransferase system	5,155	13446	3264
GO:0015612	ABC-type L-arabinose transporter activity	928	2416	2652
GO:1904680	peptide transmembrane transporter activity	2,140	5562	2777
GO:0031317	tripartite ATP-independent periplasmic transporter complex	821	2122	1883
GO:0006012	galactose metabolic process	2,736	7043	4587
GO:0006855	xenobiotic transmembrane transport	1,452	3724	3740
GO:0015675	nickel cation transport	833	2067	2256
GO:0016052	carbohydrate catabolic process	1,899	4631	1643
GO:0003690	double-stranded DNA binding	2,183	5105	-81
GO:0030420	establishment of competence for transformation	4,023	9049	581
GO:0015774	polysaccharide transport	1,076	2393	1451
GO:0008115	sarcosine oxidase activity	1,863	4134	5222
GO:0043682	P-type divalent copper transporter activity	2,571	5685	4307
GO:0042626	ATPase-coupled transmembrane transporter activity	6,621	13762	6725
GO:0008783	agmatinase activity	1,017	2076	2312
GO:0008878	glucose-1-phosphate adenylyltransferase activity	1,077	2131	1702
GO:0004556	alpha-amylase activity	1,192	2322	-102
GO:0046653	tetrahydrofolate metabolic process	2,007	3877	5070
GO:0015417	ABC-type polyamine transporter activity	1,282	2382	2781
GO:0006144	purine nucleobase metabolic process	1,187	2201	1570
GO:0055085	transmembrane transport	43,304	80099	56900
GO:0008901	ferredoxin hydrogenase activity	2,620	4773	6110
GO:0030246	carbohydrate binding	7,014	12723	9705
GO:0015833	peptide transport	18,228	31913	26432
GO:0008260	succinyl-CoA:3-oxo-acid CoA-transferase activity	1,479	2520	799
GO:0006824	cobalt ion transport	1,642	2719	2389
GO:1990961	xenobiotic detoxification by transmembrane export across the plasma membrane	2,431	4006	4295
GO:0003863	3-methyl-2-oxobutanoate dehydrogenase (2-methylpropanoyl-transferring) activity	1,422	2252	1667
GO:0006829	zinc ion transport	2,007	3072	2120
GO:0004658	propionyl-CoA carboxylase activity	2,756	4170	5815
GO:0015594	ABC-type putrescine transporter activity	3,330	4836	5379
GO:0043024	ribosomal small subunit binding	2,485	3587	3559
GO:0140359	ABC-type transporter activity	17,367	23676	17570
GO:0017168	5-oxoprolinase (ATP-hydrolyzing) activity	1,870	2540	3275
GO:0016810	hydrolase activity, acting on carbon-nitrogen (but not peptide) bonds	1,901	2566	931
GO:0071897	DNA biosynthetic process	2,601	3479	3627
GO:0008812	choline dehydrogenase activity	1,879	2414	2504
GO:0033812	3-oxoadipyl-CoA thiolase activity	1,764	2181	1925
GO:0000034	adenine deaminase activity	1,738	2148	881
GO:0019253	reductive pentose-phosphate cycle	2,806	3428	5588
GO:0006146	adenine catabolic process	1,738	2108	965
GO:0055052	ATP-binding cassette (ABC) transporter complex, substrate-binding subunit-containing	43,637	52747	56871
GO:1902494	catalytic complex	2,476	2977	3938
GO:0102483	scopolin beta-glucosidase activity	1,873	2215	500
GO:0004527	exonuclease activity	2,558	3022	1108
GO:0043419	urea catabolic process	3,698	4276	7133
GO:0009039	urease activity	3,698	4276	7133
GO:0008802	betaine-aldehyde dehydrogenase activity	4,237	4898	4388
GO:0019285	glycine betaine biosynthetic process from choline	4,838	5281	5018

GO:0006166	purine ribonucleoside salvage	2,983	3212	1109
GO:0004318	enoyl-[acyl-carrier-protein] reductase (NADH) activity	1,931	2024	2024
GO:0005978	glycogen biosynthetic process	5,173	5403	3426
GO:0016151	nickel cation binding	10,843	11252	17719
GO:0008775	acetate CoA-transferase activity	2,241	2312	428
GO:0031460	glycine betaine transport	2,364	2290	1505
GO:0019333	denitrification pathway	3,113	2929	1753
GO:0008965	phosphoenolpyruvate-protein phosphotransferase activity	2,636	2379	2305
GO:0046177	D-gluconate catabolic process	2,873	2554	1686
GO:0043190	ATP-binding cassette (ABC) transporter complex	30,202	26827	31571
GO:0004129	cytochrome-c oxidase activity	10,045	8620	5424
GO:0015031	protein transport	31,181	26638	24408
GO:0005507	copper ion binding	11,000	9044	4436
GO:0015419	ABC-type sulfate transporter activity	3,764	3093	2122
GO:0033232	ABC-type D-methionine transporter activity	3,811	3125	1881
GO:0004803	transposase activity	7,286	5968	7300
GO:0050897	cobalt ion binding	5,221	4276	5868
GO:0008556	P-type potassium transmembrane transporter activity	4,293	3488	3207
GO:0005980	glycogen catabolic process	2,606	2097	2375
GO:0046914	transition metal ion binding	3,345	2678	1614
GO:0006015	5-phosphoribose 1-diphosphate biosynthetic process	2,973	2302	1108
GO:0019563	glycerol catabolic process	3,406	2565	606
GO:0004553	hydrolase activity, hydrolyzing O-glycosyl compounds	5,554	4159	1383
GO:0006865	amino acid transport	18,697	13956	6061
GO:0019646	aerobic electron transport chain	7,145	5322	2675
GO:0004497	monooxygenase activity	4,700	3496	4908
GO:0060187	cell pole	3,274	2385	2921
GO:0070469	respirasome	7,416	5261	3565
GO:0008177	succinate dehydrogenase (ubiquinone) activity	3,402	2317	1226
GO:0015424	ABC-type amino acid transporter activity	5,249	3545	3856
GO:0016787	hydrolase activity	36,954	23813	15266
GO:0045727	positive regulation of translation	6,253	3594	3659
GO:0006108	malate metabolic process	4,072	2211	3460
GO:0016301	kinase activity (regulation of ABC transport cassete)	22,385	12097	2364
GO:0034040	ATPase-coupled lipid transmembrane transporter activity	6,780	3617	1499
GO:0016740	transferase activity	21,247	10953	-2738
GO:0004356	glutamate-ammonia ligase activity	6,303	3089	4689
GO:0016757	glycosyltransferase activity	9,607	4690	3811
GO:0000271	polysaccharide biosynthetic process	6,035	2844	2946
GO:0030956	glutamyl-tRNA(Gln) amidotransferase complex	5,174	2372	4221
GO:0003700	DNA-binding transcription factor activity	48,840	21768	12344
GO:0045892	negative regulation of DNA-templated transcription	13,359	5896	-1533
GO:0009234	menaquinone biosynthetic process	5,773	2516	-1039
GO:0004148	dihydrolipoyl dehydrogenase activity	5,072	2157	1905
GO:0016705	oxidoreductase activity, acting on paired donors, with incorporation or reduction of molecular oxygen	7,493	3180	4721
GO:0006098	pentose-phosphate shunt	6,934	2934	3528
GO:0006313	transposition, DNA-mediated	11,394	4820	5794
GO:0022857	transmembrane transporter activity	60,716	25493	13189
GO:0019752	carboxylic acid metabolic process	4,873	2021	2070
GO:0006119	oxidative phosphorylation	8,221	3402	2729
GO:0004029	aldehyde dehydrogenase (NAD+) activity	6,274	2562	2545
GO:0015930	glutamate synthase activity	4,921	2009	2003

GO:0004748	ribonucleoside-diphosphate reductase activity, thioredoxin disulfide as acceptor	8,233	3177	5894
GO:0020037	heme binding	38,623	14369	6396
GO:0005975	carbohydrate metabolic process	41,423	14756	9037
GO:0009236	cobalamin biosynthetic process	19,122	6228	8126
GO:0016616	oxidoreductase activity, acting on the CH-OH group of donors, NAD or NADP as acceptor	10,385	3284	1376
GO:0031419	cobalamin binding	14,410	4522	7233
GO:0004315	3-oxoacyl-[acyl-carrier-protein] synthase activity	10,288	3193	3291
GO:0006542	glutamine biosynthetic process	7,245	2150	4376
GO:0006071	glycerol metabolic process	8,300	2450	1197
GO:0005576	extracellular region	27,643	7568	-5170
GO:0009399	nitrogen fixation	15,641	4153	11545
GO:0035999	tetrahydrofolate interconversion	9,237	2226	1873
GO:0016491	oxidoreductase activity	55,174	13119	9757
GO:0009058	biosynthetic process	8,442	2002	3304
GO:0042910	xenobiotic transmembrane transporter activity	11,279	2645	1293
GO:0030163	protein catabolic process	8,997	2010	3209
GO:0006633	fatty acid biosynthetic process	31,634	6871	7573
GO:0006006	glucose metabolic process	11,522	2436	1633
GO:0042803	protein homodimerization activity	13,350	2351	851
GO:0030145	manganese ion binding	23,344	3856	3139
GO:0008652	amino acid biosynthetic process	17,764	2921	3764
GO:0005886	plasma membrane	685,895	110820	29476
GO:0006260	DNA replication	48,951	7268	10192
GO:0003887	DNA-directed DNA polymerase activity	26,475	3866	248
GO:0016020	membrane	63,016	9168	-2228
GO:0016310	phosphorylation	76,021	9660	-507
GO:0000166	nucleotide binding	37,879	4773	7544
GO:0008408	3'-5' exonuclease activity	23,169	2635	-633
GO:0071949	FAD binding	21,762	2436	5169
GO:0003677	DNA binding	259,969	23120	-283
GO:0006096	glycolytic process	45,041	3849	3859
GO:0005524	ATP binding	811,496	68817	94888
GO:0006508	proteolysis	74,888	4300	-12168
GO:0046872	metal ion binding	490,357	15192	13443
GO:0071555	cell wall organization	74,748	2266	-8746
GO:0050660	flavin adenine dinucleotide binding	71,846	2170	-3077
